# Proposal for determining absolute biological effectiveness of boron neutron capture therapy—the effect of ^10^B(n,α)^7^Li dose can be predicted from the nucleocytoplasmic ratio or the cell size

**DOI:** 10.1093/jrr/rry080

**Published:** 2018-11-05

**Authors:** Koji Ono, Hiroki Tanaka, Yuki Tamari, Tsubasa Watanabe, Minoru Suzuki, Shin-ichiro Masunaga

**Affiliations:** 1 Kansai BNCT Medical Center, Osaka Medical College, 2–7 Daigaku-machi Takatsuki-shi, Osaka, Japan; 2 Particle Radiation Oncology Research Center, Kyoto University, 2-Asashiro-Nishi, Kumatori-cho, Sennan-gun, Osaka, Japan; 3 Division of Radiation Life Science, Kyoto University, 2-Asashiro-Nishi, Kumatori-cho, Sennan-gun, Osaka, Japan; 4 Division of Radiation Medical Physics, Institute for Integrated Radiation and Nuclear Science, Kyoto University, 2-Asashiro-Nishi, Kumatori-cho, Sennan-gun, Osaka, Japan

**Keywords:** boron-neutron dose, D_0_, BPA, BSH, N/C ratio, cell size

## Abstract

The relationship between the radiation dose delivered to a tumor and its effect is not completely predictable. Uncertainty in the estimation of the boron concentration in a tumor, variation in the radiation sensitivity of the tumor cells, and the complexity of the interactions between the four types of radiation comprising the boron neutron capture therapy (BNCT) dose contribute to this uncertainty. We reanalyzed the data of our previous papers to investigate the variation in radiosensitivity of tumor cells to the ^10^B(n,α)^7^Li dose: the dose generated by the reaction of thermal neutrons and ^10^B, hereafter the ‘boron-neutron dose’. The radiosensitivities of five tumors (EL4, SAS/neo, SAS/mp53, SCCVII and B16-BL6 melanoma) were examined. For the combination of *p*-boron-L-phenylalanine (BPA: C_9_H_12_BNO_4_) with neutron irradiation, D_0_, the cell survival curve for the boron-neutron dose was the smallest for the SAS/neo, followed by the EL4, SAS/mp53, SCCVII and B16-BL6 melanoma, in that order. For the combination of mercaptoundecahydrododecaborate (BSH: Na_2_B_12_H_11_SH) with neutron irradiation, D_0_ was the smallest for the EL4, followed by the SAS/neo, B16–BL6melanoma, SAS/mp53 and SCCVII, in that order. The relationships between these D_0_ values and the nucleocytoplasmic ratios (Xncs) or cell size indices (Xcs) obtained by histopathological microslide image were as follows: (D_0_ = 0.1341Xnc^–1.586^, *R*^2^ = 0.9721) for all tumor types with BPA-BNCT, and D_0_ = 0.0122Xcs–0.1319 (*R*^2^ = 0.9795) for four tumor types (all except the B16-BL6 melanoma) with BSH-BNCT. Based on these results, we proposed a new biologically equivalent effectiveness factor: the absolute biological effectiveness (ABE) factor. The ABE factor is Gy/D_0_. Thus, the ABE dose is the physical dose multiplied by the ABE factor, and refers to the dose needed to decrease the cell survival rate to e^–ABE dose/Gy^.

## INTRODUCTION

Slow-speed neutrons (thermal neutrons) are captured by the boron isotope ^10^B nucleus with an incredibly high probability, compared with capture by other elements comprising the body, and they immediately split the nucleus into an α particle and a lithium (Li) nucleus: ^10^B(n,α)^7^Li. These particles have maximum ranges of 9 μm and 4 μm, respectively, which do not exceed the diameter of most cells. They release all their kinetic energy along their tracks, so they are high–linear energy transfer (LET) radiation and their relative biological effectiveness (RBE) is extremely high. The dose generated by the reaction of thermal neutrons and ^10^B is hereafter the ‘boron-neutron dose’. Oxygen pressure doesn’t affect the cell-killing effect of these particles. Since the biological effect of the irradiated thermal neutrons themselves is small, a selective killing effect at cell level can be expected if a ^10^B-based drug selectively accumulates in tumor cells. Cancer therapy using these principles is known as boron neutron capture therapy (BNCT).

In clinical studies using research reactors, promising results have already been reported in the treatment of malignant brain tumor [1–6], malignant melanoma [7–10], head and neck cancer [11–15], and other cancers. Currently, the use of *p*-boron-L-phenylalanine (BPA:C_9_H_12_BNO_4_) is being extensively studied in clinical research. Mercaptoundecahydrododecaborate (BSH: Na_2_B_12_H_11_SH) is also being used, mainly for the treatment of malignant brain tumors [1, 16]. From the clinical results of BNCT using these boron drugs, however, the relationship between the radiation dose applied to the tumor and its effect is not necessarily clear [4, 17, 18]. Researchers into BNCT are struggling to properly evaluate the dose required, and a dose evaluation method that can accurately relate the dose to its effect is needed. Using the conventional method, the simple sum of the biologically equivalent dose [obtained by multiplying each dose component by the fixed value of the relative biologic effectiveness (RBE)], otherwise known as the ‘compound biological effectiveness’ (CBE), is taken as the total dose. However, in this case, the dose becomes unrealistically large [19].

In order to solve this problem, in 2012 a method for constructing a realistic dose – cell survival curve was developed by incorporating the primary repair of sublethal damage and the synergistic effect between each component of high-LET radiation and the gamma rays into the dose–survival curve of malignant melanoma cells [19]. By comparing the BNCT dose–survival curve thus determined with the dose – cell survival curve for a photon beam, the photon-isoeffective (IsoE) dose was obtained. This IsoE dose was much lower than the fixed RBE method dose, and more accurately represented the quantitative relationship between the tumor dose and the response of the malignant melanoma lesion. In 2017, this dose assessment method was applied to the analysis of patients with head-and-neck tumors being treated with BNCT, and its usefulness in the prediction of tumor response and occurrence of mucositis was confirmed [20]. In both analyses, it was assumed that the ratio of the ^10^B level in tumor cells to that in normal tissue (blood) is a fixed value. However, variation in the ^10^B level ratio in individual patients, and variation in the ^10^B drug distribution within a tumor, should always be taken into account. Another point that must be considered is between-tumor variation in the cell-killing effect of the boron-neutron dose at the cellular level.

Since the LETs of particles released in the boron neutron capture reaction are extremely high, in the ‘overkill region’ in relation to RBE, there may be less variation in the cell-killing effect between tumors than occurs during X-ray irradiation [21]. However, we decided it was necessary to confirm whether this is true and reanalyzed our previously published data on experimental tumor models, searching for the factors that determine the response of each tumor type to the boron-neutron dose.

## MATERIALS AND METHODS

### Analysis of the relationship between the boron-neutron dose and the cell-killing effect

In our earlier work, the SCCVII tumor, B16-BL6 melanoma, EL4 leukemia, and human oral cancer tumors SAS/neo and SAS/mp53 (in which a mutant *p53* gene was introduced into *SAS*/*neo*) in mice were irradiated with neutron beams in combination with BPA or BSH [22–25]. After irradiation, the tumors were removed from the mice, and a single cell suspension was made by enzymatic digestion. Next, the isolated tumor cells were plated onto petri dishes and their survival rates were determined by colony-formation assay. All neutron beam irradiations were performed using the heavy water facility of the Kyoto University Research Reactor (KUR). The energy spectra of the irradiating neutron beams differed between experiments. In some experiments, a pure thermal neutron beam was used, but in others a mixed beam of thermal neutrons, epithermal neutrons and fast neutrons was used. Fortunately, the neutron beam alone without boron drugs was used in every experiment, so the effect of the boron-neutron dose to cells in the tumor was able to be extracted by using this experimental data as a control. The reanalysis procedure is described in detail as follows.

In our earlier work, the cell survival rate was plotted against the thermal neutron fluence (n/cm^2^) in the SCCVII tumor experiment, but in the experiments with the other types of tumor the cell survival rateswere shown in relation to the total radiation dose: the sum of the boron-neutron dose, the nitrogen-neutron dose [^14^N(n,p)^14^C (the dose generated by the reaction of the thermal neutron and ^14^N)], the epithermal neutron dose, the fast neutron dose [^1^H(n,n’)^1^H] and the γ-ray dose. The dose component from the reaction caused by epithermal neutron with ^10^B or ^14^N is quite small compared with that of other components (i.e. <1.4% of the total dose even at 10 ppm of ^10^B). When ^10^B does not coexist, the proportion of these dose components is determined depending on the energy spectrum of the neutron beam used. Therefore, the biological effect (cell survival rate) of the neutron beam used can be determined from a function of the thermal neutron fluence. When ^10^B is present, the boron-neutron dose is added to this, but the cell survival rate is likewise a function of the thermal neutron fluence. The decrease in the cell survival rate due to the coexistence of ^10^B corresponds to the cell-killing effect of the boron-neutron dose.

**Fig. 1.**
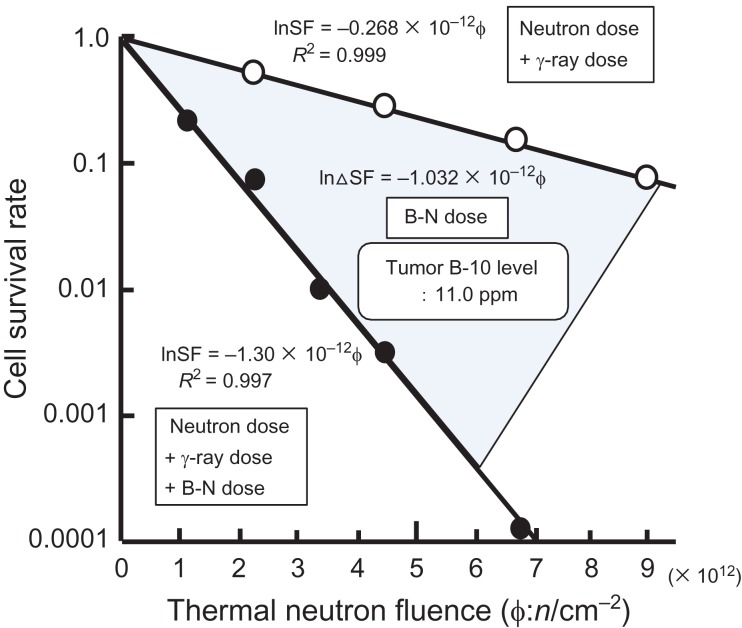
Method for determining the D_0_ of the boron-neutron dose. This figure was created based on the data of Ono *et al.* [22].

According to this hypothesis, the relationships between tumor cell survival rate curves and thermal neutron fluencewere rewritten using the data obtained by neutron irradiation alone as a control. It was found that the tumor cell survival rate after neutron beam irradiation decreased exponentially with increase in the thermal neutron fluence with or without ^10^B. Then, the influence of boron-neutron dose on the survival rate of the tumor cells was obtained from the difference between the slopes of the two exponentially decreasing straight lines. The subtracted cell survival rate also decreased exponentially with respect to the thermal neutron fluence. The neutron fluence necessary to lower the cell survival rate by e^–1^ on the subtracted exponentially decreasing line was defined as ϕ_0_, similar to D_0_ (Fig. 1). The D_0_ of the boron-neutron dose was calculated based on the ϕ_0_ thus obtained, and the ^10^B concentration in the tumor as follows:

$$D_{0}=\phi_{0}\times 6.933\times 10^{–14}\times^{10}B\;\mathrm{concentration}\;\left(\mathrm{ppm}\right).$$

### Determining the nucleocytoplasmic ratio and cell size index on the microscopic image of the histopathological slide

For this research, we used the Keyence BZ-8000 microscope, which has an inbuilt image analysis system. A region avoiding the interstitial component as much as possible was selected on the image of the tumor histopathological slide subjected to hematoxylin and eosin (HE) staining and its area was measured. After that, using the image analysis software, the area occupied by the cytoplasm was determined by color tone; its area was also measured. The area obtained by subtracting the latter from the former is the area occupied by the nuclei.

**Fig. 2.**
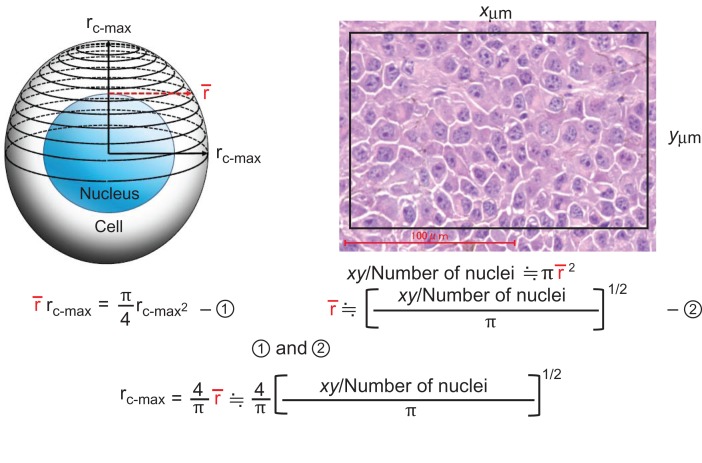
Method for estimating cell diameter.

Tumor cells are randomly arranged in the tissue. Because the histopathological slide is a random cross-section of their nuclei and cytoplasm, the ratio of the total area of the nuclei to the initially selected area is the nucleocytoplasmic (N/C) ratio. Similarly, when the number of nuclei in the selected area is counted and the area is divided by the number of nuclei, the value (μm^2^) becomes an indicator of the cell size. Naturally, this is not equal to the cell area based on the largest diameter. However, an equation for estimating the radius of the great greatest diameter of the cell from this cell size index is provided (Fig. 2). Next, the nucleus diameter can be estimated from the estimated cell diameter and the N/C volume ratio obtained as described.

### Determining the nucleus and cell diameters in cultured cells

Five kinds of tumor cells were cultured in the medium described in papers of our earlier work, cells were harvested in the logarithmic growth phase, and a cell suspension was prepared. After diluting the solution of the fluorescent reagent Hoechst 33 342 for staining live cell nuclei, this was added to the suspension and it was placed in a carbon dioxide incubator for 15 min. The culture medium containing the fluorescent reagent was replaced with PBS by centrifugation for microscope observation. The cell suspension, in which the cell nuclei were stained with Hoechst 33 342, was placed on the blood cell counting board and a brightfield image was acquired. Also, a fluorescent microscope image of the same area was obtained. Using a ruler imprinted on these images, the cell diameter and nucleus diameter were measured for >100 cells.

## RESULTS

D_0_ versus total dose was presented in a cell survival curve under various irradiation conditions and compared for five kinds of tumors. There was no consistent tendency regarding trends in the magnitude of D_0_. The tumor type showing the minimum or maximum value of D_0_ varied depending on whether it was treated with a neutron beam alone or a neutron beam in combination with any boron drug (Table 1).

**Table 1.** D_0_ of total dose (Neutron + BPA + BSH)

Tumor	D_0_ (Gy)		
	Neutron beam	BPA + neutron beam	BSH + neutron beam
EL4	0.98	0.52	0.81
B16–BL6	1.87	1.16	1.34
SAS/neo	2.42	0.41	1.27
SAS/mp53	2.51	0.6	1.57
SCCVII	2.25	1.36	2.55

Table 2 presents the data for the D_0_ versus the boron-neutron dose in a cell survival curve in which BPA or BSH were combined with the neutron beam. A variation of >2-fold was observed within both BPA-BNCT and BSH-BNCT, depending on tumor type. Table 3 shows the N/C ratio and the cell size index of five tumors examined in histopathological specimens, assuming a relationship with D_0_. EL4 had the smallest cell size index and the largest n/c ratio, which had been expected from the microscope image. In addition, the cell size of the B16-BL6 melanoma was the largest, and its N/C ratio was the smallest. This was also as expected from the microscope image. SAS/neo and SAS/mp53 originated from the same cell line; however, there existed definite differences between them.

**Table 2.** D_0_ of boron-neutron dose

Tumor	^10^B(n,α)^7^Li dose D_0_ (Gy)	
	BPA	BSH
EL4	0.43	0.75
B16–BL6	0.91	1.25
SAS/neo	0.35	1.2
SAS/mp53	0.52	1.44
SCCVII	0.74	1.6

**Table 3.** Nucleocytoplasmic ratio and cell size index for five tumors

Tumor	N/C volume ratio	Cell size index (μm^2^)
EL4	0.508	74.5
B16-BL6	0.3	202
SAS/neo	0.528	108
SAS/mp53	0.407	123
SCCVII	0.347	146

Table 4 compares the cell diameters and the nuclear diameters obtained from the histopathological specimens with those observed in cultured cells. On the whole, a very clear relationship can be seen. However, in the B16-BL6 melanoma, which has a low N/C ratio, both the cell size and the nuclear diameter were overestimated from the histopathological slide. The degree of agreement was very good for EL4, which has a large N/C ratio.

**Table 4.** Comparison of estimated and measured diameters between *in vivo* and *in vitro* conditions

Tumor type	Diameter of nucleus (μm)		Diameter of cell (μm)	
	*In vivo*	*In vitro*	*In vivo*	*In vitro*
EL4	9.8	9.7	12.3	12.5
B16-BL6	13.6	11.9	20.4	18.4
SAS/neo	12.1	11.6	14.9	14.9
SAS/mp53	11.8	12.8	15.9	16.2
SCCVII	13.0	13.6	18.5	16.8

**Fig. 3.**
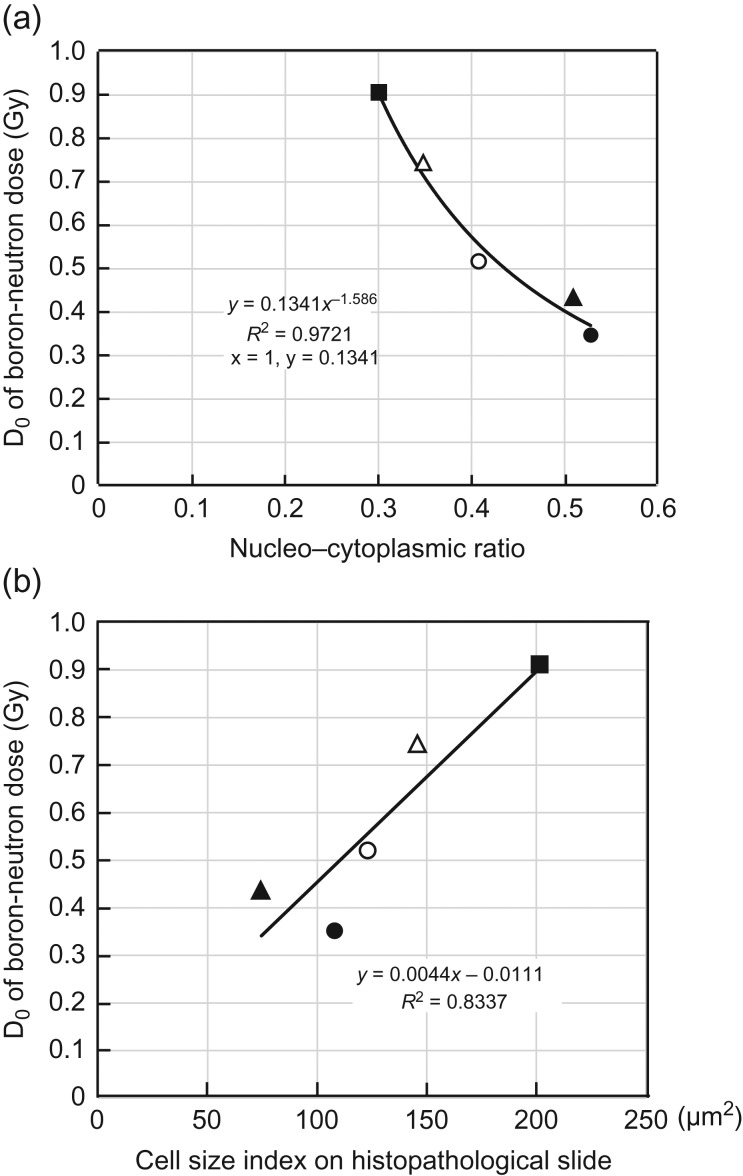
(a) BPA-D_0_ versus nucleocytoplasmic ratio. (b) BPA-D_0_ versus cell size index.

Figure 3a shows the relationship between the boron-neutron dose D_0_ in BPA-BNCT and the N/C ratio (Xnc) of the cells. As the ratio increased, D_0_ decreased. There was a very strong correlation between the two in the power approximation (D_0_ = 0.1341Xnc^–1.586^, *R*^2^ = 0.9721). There existed a correlation between the cell size index (Xc) of each tumor and the D_0_ of the boron-neutron dose in their BPA-BNCT by linear relationship (Fig. 3b).

**Fig. 4.**
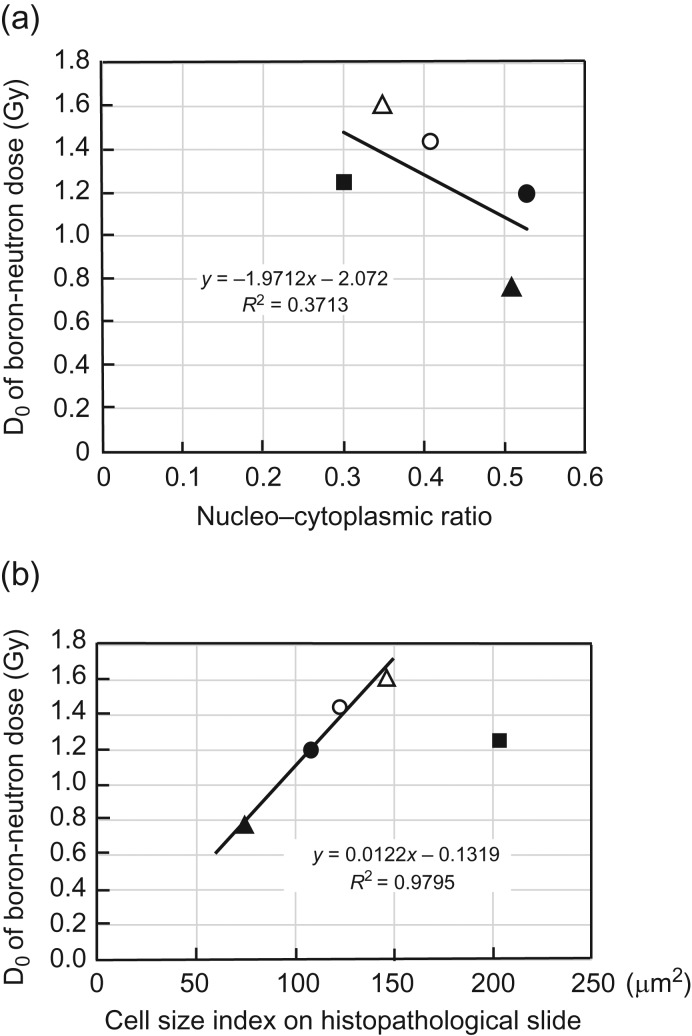
(a) BSH-D_0_ versus nucleocytoplasmic ratio. (b) BSH-D_0_ versus cell size index.

Figure 4a shows the relationship between the D_0_ of the boron-neutron dose in the BSH-BNCT and the Xnc of the cell. No correlation was found between the two parameters. However, D_0_ increased with increasing Xcs, except for the B16-BL6 melanoma (Fig. 4b). When restricted to four tumors, a very strong correlation was found in the linear relationship between D_0_ and Xcs (D_0_ = 0.0122Xcs–0.1319, *R*^2^ = 0.9795). The B16-BL6 melanoma, however, greatly deviated from linear relationship.

## DISCUSSION

We investigated whether the radiosensitivity of tumor cells to the boron-neutron dose differed between tumor types and whether this might explain why the dose–effect relationship in clinical data is not clear.

### Analysis of total dose versus cell survival, comparing neutron irradiation alone with BNCT

The radiosensitivity of tumor cells to neutron radiation alone or to neutron radiation in combination with boron drugs was reanalyzed for three experimental animal tumors and two human-derived tumors. As shown above, there was a large difference in D_0_ in the dose – cell survival curves between tumor types (Table 1). Although another was an *in vitro* study, we found that there was no difference in radiation sensitivity to pure thermal neutron beam irradiation between four of the types of brain tumor cells, which are known to be significantly different in sensitivity to gamma rays [26]. In the experiment used for this reanalysis, the energy spectrum of the neutron beam differed from experiment to experiment. This means that a difference existed in the mixing ratio between the high-LET neutron component and the low-LET primary and secondary γ-ray components in each experiment. This difference between the mixing ratio may be the reason for the large differences in the radiosensitivity of the various tumors to the neutron beam alone. In addition, the tumors were irradiated under *in vivo* conditions in the experiments used for reanalysis. It is inferred that this may also be another reason for obtaining different results.

The large variation in radiation sensitivity found between tumors when neutron beam irradiation was combined with a boron drug is thought to be attributable to the fact that the mixing ratio of the thermal neutron component inducing the ^10^B(n,α)^7^Li reaction varied, and also to the fact that the tumor ^10^B levels differed between studies [22–25].

### Cell survival curves of tumors to according to boron-neutron dose (determining the D_0_ value) for the various tumor types and boron drugs

In order to investigate the pure radiosensitivity of each tumor to the boron-neutron dose, we applied the analysis operation illustrated in Fig. 1. Usually, another method is used. First, four kinds of doses constituting the BNCT dose are separately calculated. The dose–survival curve for the boron-neutron dose is then obtained by stepwise subtraction of the influence of each dose component from the total dose–survival curve. However, it is quite doubtful whether it is possible to completely separate the effect of the whole beam of mixed high-LET radiation and low-LET radiation into the effect of each dose component [19, 27].

Looking at the extracted dose – cell survival curve in this study, the D_0_ value varied by more than 2-fold between the tumors for both BPA- and BSH-BNCT. This was an unexpected surprise. Both α particle and Li nucleus radiations are high-LET radiations (in an overkill region beyond the peak of RBE). The oxygen enhancement ratio is 1.0, and the oxygen pressure around the tumor cell doesn’t have any effect on cell killing by these particles [21]. Therefore, this phenomenon was raised as a new problem to be solved.

### Consideration of influence of N/C ratio or cell size on D_0_ value

Since DNA double-strand breaks are directly linked to cell death, the possibility that the dose given to the nucleus of the cell differs depending on the tumor needs to be considered, even if the doses at the macro level are equal. The unit of D0 is Gy, which is a unit representing energy deposition defined on macroscopic scale by joule/kilogram. The ranges of both α particles and Li nuclei are <10 μm. If the reaction occurs in the nucleus, both particles can cause double-strand breaks in DNA; however, due to the relationship between the direction of the particle and the position of the nucleus, only one of two particle, can induce DNA cleavage in the case of reaction in the cytoplasm. Also, depending on the location of the reaction, neither particle may be able to reach the nucleus from the point of distance and direction. Thus, reactions occurring in the cytoplasm may not be sufficient to cause cell death in terms of either distance and direction. In general, however, the volume of the cytoplasm is larger than the volume of the nucleus, so there are more ^10^B atoms present there. Therefore, it may be possible that boron-neutron reactions generated in the cytoplasm generally induce more DNA damage.

In addition, it is necessary to consider the following points. The LET value of a particle changes depending on its position on the track. When the boron-neutron reaction occurs in the nucleus, the DNA damage is induced by particles with extremely high LET. However, as mentioned earlier, their particles cause excessive DNA damage compared with the amount necessary cause cell death, so cell death is not proportionate to the dose from the particles. Thus, it can be imagined that the effect of BNCT is determined quantitatively by the boron-neutron reaction occurring in both the nucleus and cytoplasm. This is the case when the boron drug has accumulated in the cytoplasm or nucleus. On the other hand, when most of the boron drug remains outside the cell or on the cell membrane, the cell-killing effect seems to be determined by the cell size. It is assumed that the cell-killing effect remarkably decreases as the cell size increases. Of the two boron drugs, BPA is known to be taken up by cells and uniformly distributed throughout the cytoplasm and nucleus. Although the behavior of BSH may vary depending on the cell type, it is distributed in the intercellular space and it is generally considered difficult for it to enter into the cell.

For determining the bioequivalent boron-neutron dose in BNCT, it is indispensable to consider the microscopic distribution of the dose, that is, the geometrical microscopic distribution of the boron drug.

The N/C ratio of each tumor and D_0_ of the boron-neutron dose in their BPA-BNCT cell survival curves have a very strong correlation in power approximation (Fig. 3a). When considering what kind of approximate curve is optimum, a strong correlation equivalent to the power approximation is also indicated from the decision coefficient (*R*^2^) in linear approximation, exponential approximation, logarithmic approximation and polynomial approximation. But, in any approximations other than power approximation, when the N/C ratio approaches 1.0, an unnatural D_0_ value (i.e. sometimes a negative or too large a value) is predicted. Therefore, power approximation was adopted. There was a linear correlation between the Xcs of each tumor and the D_0_ of the boron-neutron dose for BPA-BNCT (Fig. 3b). Although the correlation degree was the highest among various approximations, it was still much lower than the correlation degree when D_0_ was approximated according to the N/C ratio. This result shows that the N/C ratio is more accurate for predicting the D_0_ of the boron dose for BPA-BNCT.

For BSH-BNCT, a correlation did not exist between D_0_ of the boron-neutron dose and Xnc (Fig. 4a), but there was an accurate linear correlation between D_0_ and the Xcs except for with B16-BL6 melanoma (Fig. 4b). This result shows that the Xcs is useful for predicting the D_0_ of the boron dose in BSH-BNCT, with some exceptions. This would indicate that the microdistribution of BSH in a B16 melanoma could be different from that in the other four tumor types. It is also reported that the accumulation of BSH on the cell greatly varies depending on the cell type. In the report by Capala in 1996 [28], when B16 melanoma cells were incubated with BSH for a long period (18 h), the accumulation concentration did not differ from that when incubated with BPA. In our experiments, the contact time was much shorter, but the same observation was made.

In order to explain the data obtained in this reanalysis, we calculated the radiation energy imparted to the nucleus in BNCT. In calculations, the physical dose must be well correlated with the biologic effect. Therefore, we devised two new radiation energy concepts incorporating the biologic effects: they are termed RBE-LET and RBE-keV, which are LET and keV multiplied by RBE, respectively. The calculated bioequivalent energies imparted to the nucleus using these new concepts were in good agreement with the radiation biological analysis reported in this paper. Currently, we are writing a paper about this physical calculation.

In BNCT, it has long been claimed that the dose imparted to the nucleus and cytoplasm by particles, and thus the distribution of boron, greatly influence the biologic effect [29, 30]. The microdosimetry model, which is capable of calculating the surviving fractions, RBE values and boron concentration distributions, has been reported. By using this model, the surviving fractions were generated for V79 Chinese hamster cells. The measured surviving fractions in experiments agreed well with these fractions calculated by the microdosimetry model, within the uncertainties of the measurements [31].

Since macrodosimetry could not explain the apparent differences in clinical outcome between tumor types, a computer program was created, providing an improved tissue model for microdosimetry techniques. This model permitted the dose in each cell’s cytoplasm and nucleus, and the interstitium to be calculated for ellipsoidal cells placed in either random or ordered locations. According to this model, the difference in clinical outcome was reported to arise from the tissue’s cellular geometry and the effects of neighboring cells [32]. In research, since 3D information at the cellular level was ideal for the biological effect study of BNCT, a novel microdosimetry analysis was applied to the autoradiography of an individual human glioblastoma multiforme and normal brain, and the specific energy distribution was obtained directly. The combination of this microdosimetry analysis with a biophysical model for cell survival analysis based on the specific energy was able to predict cell survival curves for uniform and non-uniform ^10^B distributions. The results indicated that the dose–survival curve varied widely between tumors. This finding has very important clinical significance [33].

Our current work clearly demonstrates that individual tumor responses to the boron-neutron dose, ie D_0_ in the actual dose – cell survival curve, are accurately predicted by the tumor cell nucleocytoplasm ratio and the cell size. This is similar to the variation in tumor response to BNCT speculated in Lu and Kiger’s research [33]. Furthermore, since the index can be obtained from a histopathological slide, our finding is considered to be extremely useful in BNCT.

### Validity in estimating the N/C ratio and the cell size of tumor cells based on histopathological slide images

The N/C ratio obtained from a histopathological slide is considered to be theoretically accurate. On the other hand, it is easy to see that the nucleus may not be contained in the cross-section of the cell in the case of a small N/C ratio (Fig. 3). Since the area of the selected region is divided by the number of nuclei to estimate cell size in our analysis, the risk of overestimation of cell size increases with decrease in N/C volume ratio. This risk was recognized in the data in Table 3. In B16-BL6 melanoma, the N/C ratio is small, so the cell diameter was overestimated, and this led to an overestimation of the nuclear diameter.

### Proposal of new bioequivalent dose: absolute biologic effectiveness dose

Since the radiosensitivity of tumor cells to a boron-neutron dose in BNCT is uniquely determined, it is not necessary to use the coefficients RBE and CBE as defined by the ratio to the radiosensitivity to X-ray dose. As the X-ray sensitivity of an individual patient’s tumor cells can’t be determined, typical fixed values obtained by basic experiments are usually used. Therefore, RBE-Gy and CBE-Gy are never precise bioequivalent doses for individual tumors, but should be referred to as convenient values.

Based on the results obtained in this study, we devised a new coefficient called the absolute biologic effectiveness (ABE) factor. Its definition is ABE factor = Gy/D_0_. Since the unit of D_0_ is Gy, the ABE factor is an unitless number. The ABE dose is obtained by multiplying this coefficient by the physical dose. This ABE dose means the dose that can reduce the cell survival rate to e^–ABE dose/Gy^. If the accumulated clinical data are analyzed with this bioequivalent dose, in future, the clonogenic cell number (stem cell number) *in vivo* may be estimated for each individual patient’s tumor.

For using the ABE dose, it is necessary to precisely predict the cell-specific D_0_. Therefore, it can currently only be used in BNCT that emits extremely high-LET particles.

### Research subjects to make ABE dose clinically usable

This report is a study using experimental tumor models with only a few interstitial components. Furthermore, the macroscopic necrosis or bleeding part was removed from the sample for the measurement of ^10^B concentration. Therefore, it can be assumed that the boron concentration was extremely close to the concentration in the tumor cell mass. However, in the clinical situation, this non-tumor component is included in the voxel of the PET image, and there is a possibility that the ^10^B concentration estimated by PET may be lower than the actual concentration. Even if the concentration in the tumor cell mass is the same, it may be observed as a different concentration that varies depending on the properties of the tumor tissue. This will not be easy to resolve, but I look forward to an improvement in our understanding with developments in PET technology and the combination of imaging methods such as PET and MRI.

## CONCLUSION

A very strong correlation was found between the boron-neutron dose D_0_ and the N/C ratio or the cell size index. Thus, according to the equation derived in this paper, it was possible to accurately predict D_0_ for individual patient tumors from a histopathological slide prior to BNCT. Although FBPA PET is indispensable in determining how much boron drug actually accumulates in individual tumors, we believe that the findings of this study will contribute to our general understanding of BNCT and improve the accuracy with which it can be applied.
